# Three-Dimensional Printing Biomimetic Ceramic Composites Inspired by the Desert Scorpion with Excellent Erosion Wear Resistance

**DOI:** 10.3390/biomimetics11040248

**Published:** 2026-04-04

**Authors:** Zhaozhi Wang, Weicong Wang, Xinhui Duan, Xu Bai, Zhibin Jiao, Chenliang Wu, Jing Zhao, Zhihui Zhang

**Affiliations:** 1 School of Mechanical Engineering, Shenyang University of Technology, Shenyang 110870, China; 746288852@smail.sut.edu.cn (W.W.); duanxinhui@smail.sut.edu.cn (X.D.); baix@sut.edu.cn (X.B.); jiaozhibin@sut.edu.cn (Z.J.); 2 School of Materials Science and Engineering, Shenyang University of Technology, Shenyang 110870, China; mfclwu@sut.edu.cn; 3 The Laboratory of Bionic Engineering (Ministry of Education), Jilin University, Changchun 130022, China; zhzh@jlu.edu.cn; 4 Institute of Structured and Architected Materials, Liaoning Academy of Materials, Shenyang 110167, China

**Keywords:** 3D printing, ceramic composites, biomimetic erosion resistance, curved-surface structure, rigid–flexible coupling

## Abstract

Inspired by the erosion-resistant dorsal armor of the desert scorpion, this study developed biomimetic ZTA ceramic composites with enhanced resistance to solid particle erosion. Three biomimetic configurations, namely convex-bump (CH-O), convex-curved-surface (CH-CS), and convex hybrid rigid–flexible (CH-HS) structures, were fabricated by direct ink writing (DIW) 3D printing. Their erosion performance was evaluated by gas–solid two-phase erosion tests at impact angles ranging from 15° to 90°, and the underlying mechanisms were elucidated through erosion morphology analysis, actual impact angle analysis, and stress-wave propagation analysis. The results showed that the erosion rate of all samples first increased and then decreased with increasing impact angle, reaching a maximum at around 60°. Compared with the smooth control sample, CH-O exhibited lower erosion resistance under low-angle erosion conditions but showed clear improvement under high-angle erosion conditions, with the erosion resistance increased by 18.39–32.54%. CH-CS further improved the erosion resistance of CH-O, with enhancements of 14.31–53.92% at low impact angles and 24.57–35.17% at high impact angles. Among all the biomimetic designs, CH-HS exhibited the best overall erosion resistance, showing an additional improvement of 9.22–32.16% over CH-CS across the tested impact angle range. The superior erosion resistance was attributed to the synergistic effects of convex-bump morphology, curved-surface-induced particle deflection, and rigid–flexible coupling. These biomimetic features modified the actual impact angle of the particles, deflected their trajectories, reduced direct particle impact, and generated a shadow effect, while the flexible layer dissipated impact energy through reflection unloading at the rigid–flexible interface. This study provides a novel strategy for the biomimetic design of erosion-resistant ceramic composites and offers new insights into mitigating erosion damage in ceramic-based mechanical components.

## 1. Introduction

In industrial production, wear causes severe damage to mechanical surfaces, and wear-induced material failure has become one of the three major modes of failure [1]. According to conservative estimates, more than 50% of machine component failures are attributed to wear, and erosion wear accounts for approximately 8% of wear-related failures in engineering applications. Strategies for improving wear resistance and erosion resistance have been extensively explored in metals and polymers [2,3], whereas relatively limited attention has been paid to erosion-resistant ceramic composites.

Ceramic composites have become critical materials for manufacturing key components in advanced equipment across fields such as aerospace, national defense, and biomedical engineering owing to their high strength, exceptional hardness, excellent wear resistance, and superior high-temperature performance [4,5]. As a result, they have attracted considerable research interest [6,7,8,9]. Among these key components, erosion wear is one of the most common forms of wear-induced failure. For instance, components such as fan blades and vectored nozzles in the vectored turbofan engines of highly maneuverable fighter aircraft, as well as rocket and missile nose cones, aerospace vehicle hulls, rotorcraft propellers, and naval propeller blades, are often susceptible to failure caused by high-speed solid particle erosion during operation. This significantly compromises the overall service life of the equipment [10]. However, as the service conditions and operating environments of such high-precision equipment become increasingly extreme and complex, the limitations of traditional erosion-resistant solutions have become more apparent and can no longer meet current demands. For example, the introduction of reinforcing phases or novel materials [11,12] may weaken the interfacial bonding within the ceramic matrix. Although ceramic coatings [13,14] can improve erosion resistance, they also exhibit inherent drawbacks such as short service life, unstable performance, and poor adhesion, making them inadequate for more severe and complex service conditions. Therefore, the development of novel erosion-resistant ceramic composites is of great theoretical and scientific significance for improving the operational stability of key components in advanced military and aerospace equipment.

In nature, desert-dwelling organisms, such as desert scorpions, desert lizards, and tamarisk—have evolved surface organs or tissues with exceptional erosion resistance to adapt to harsh environmental conditions. These specialized biological materials not only mitigate wind and sand erosion in the organisms themselves, but also provide valuable inspiration for the design of high-performance erosion-resistant functional materials for engineering applications [15,16,17,18]. For example, Yu [19] combined the convex surface morphology of the desert scorpion with a phyllotactic biomimetic arrangement to develop a novel coupled biomimetic erosion-resistant sample. This study elucidated the effects of the arrangement pattern and structural density of the biomimetic features on erosion wear. The experimental results demonstrated that the coupled biomimetic sample exhibited the best erosion wear resistance. Inspired by the desert scorpion, Han [20] developed a novel biomimetic functional surface for erosion resistance by incorporating three structural elements: convex-bumps, grooves, and curvature. The experimental results showed that the biomimetic samples exhibited superior resistance to solid particle erosion compared with conventional smooth samples. Both the convex protrusions and curved surfaces were found to enhance erosion resistance by altering the impact angle of particles striking the sample surface. Zhang [21] extracted the erosion-resistant morphological features of the desert scorpion and tamarisk, and conducted a parallel optimization design incorporating their shared characteristics—convex bumps, grooves, and heterogeneous materials—to construct a morphology–material coupled biomimetic model. Erosion tests demonstrated that the biomimetic sample integrating grooves, convex structures, and heterogeneous materials exhibited outstanding erosion resistance, with the erosion resistance improved by up to 50.96%. The aforementioned studies have demonstrated that mimicking biological features of desert scorpions, tamarisk, and other organisms—such as convex bump morphologies, curved surface structures, and rigid–flexible material properties—can effectively enhance the erosion resistance of the target specimens. However, current applications have primarily focused on metallic and polymeric materials, with limited research on coupled biomimetic erosion-resistant strategies for ceramic composites. Therefore, a deeper understanding of the erosion-resistant mechanisms of typical biological materials, together with their integration with the intrinsic characteristics of ceramic composites, could facilitate the development of novel multi-factor coupled biomimetic models with improved compatibility. This approach is expected to provide new insights into the design of advanced biomimetic erosion-resistant ceramic composites.

Owing to the inherent chemical bonding characteristics of ceramic materials, conventional manufacturing techniques such as open-mold casting and mechanical machining are often inadequate for the rapid and efficient fabrication of ceramic composites with complex biomimetic structures. Hence, there is a compelling need to explore advanced fabrication processes tailored to ceramic materials. 3D printing represents a class of disruptive manufacturing technologies that has undergone rapid development over the past three decades. Its emergence has provided a viable approach for the shaping of ceramic materials [22,23]. Currently, 3D printing techniques suitable for ceramic materials primarily include direct ink writing (DIW) [24], stereolithography (SLA) [25], digital light processing (DLP) [26], fused deposition modeling (FDM) [27], and selective laser sintering (SLS) [28]. Among these 3D printing technologies, DIW stands out as a manufacturing process characterized by low costs and environmental friendliness [29]. Its capability to fabricate engineering components with high aspect ratios and high solid loading [30] makes it one of the advanced techniques suitable for producing ceramic samples with complex biomimetic erosion-resistant structures.

Building upon the aforementioned research, this paper proposes a novel erosion-resistant strategy for ceramic composites. Guided by a multi-factor coupled biomimetic theory integrating the erosion-resistant convex-bump morphology, curved surface structures, and rigid–flexible graded material properties of the desert scorpion dorsal plate, a biomimetic erosion-resistant model was designed. Using alumina ceramic composites as the substrate, biomimetic erosion-resistant ceramic composites were fabricated by DIW 3D printing. The erosion behavior of the biomimetic samples under various impact angles was systematically investigated. By integrating the experimental results, erosion morphology analysis, actual impact angle characteristics, and stress wave propagation mechanisms, the erosion-resistant mechanisms of different biomimetic samples were thoroughly discussed. This study is expected to advance both the theoretical understanding of the erosion resistance of novel ceramic composites and the design and fabrication of biomimetic erosion-resistant specimens.

## 2. Materials and Methods

### 2.1. Experimental Materials

The experimental material used in this study was a laboratory-fabricated ZTA ceramic composite. The material system primarily consisted of ceramic powders and a premixed solution. The ceramic powders included Al_2_O_3_ powder with an average particle size of 1 μm (Anhui Estone Material Technology Co., Ltd., Hefei, China) and 8Y-TZP powder with a particle size of 500 nm (Shanghai Zhongye New Materials Co., Ltd., Shanghai, China). The premixed system consisted of a solvent and several chemical reagents. The solvent was laboratory-prepared distilled water. Ammonium polyacrylate (Foshan Shengchuangda Chemical Co., Ltd., Foshan, China) was used as the dispersant, sodium hexametaphosphate (Tianjin Hengxing Chemical Reagent Manufacturing Co., Ltd., Tianjin, China) served as the binder, and sodium alginate (Shanghai Yi En Chemical Technology Co., Ltd., Shanghai, China) was employed as the thickener. All chemical reagents were commercially sourced.

**Figure 1:**
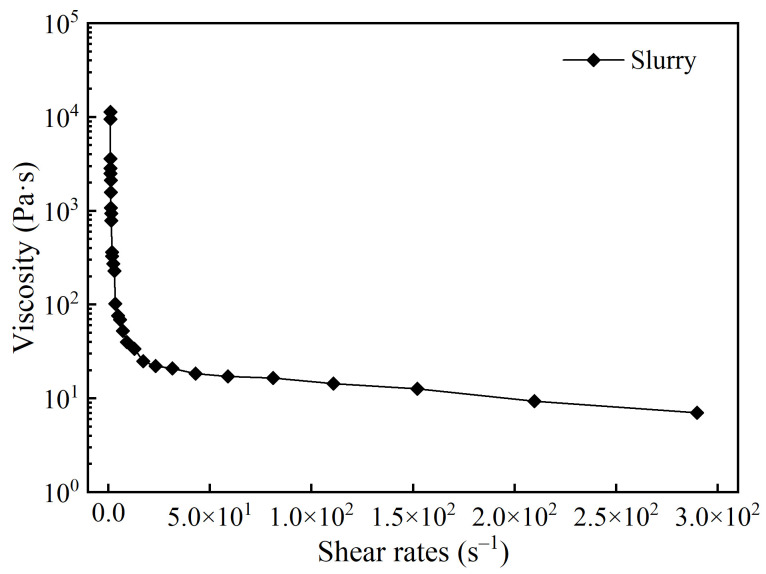
Shear rheology properties of ceramic slurry.

First, the dispersant, binder, thickener, and solvent were weighed at 1.6 wt.%, 2.4 wt.%, 0.3 wt.%, and 17 wt.% of the total mass of the ceramic powders and additives, respectively. They were then placed in a ZD-T600C planetary mixer (Shenzhen Zhidi Technology Co., Ltd., Shenzhen, China) and mixed at 600 rpm to obtain a ceramic premixed solution. Second, alumina powder and zirconia powder were weighed at a mass ratio of 2:1. After stirring, the powders were gradually added to the premixed solution and thoroughly mixed in the planetary mixer at 800 rpm. After mixing, the ceramic slurry had a solid loading of approximately 78.7 wt.%. The prepared slurry exhibited shear-thinning behavior, meeting the requirements of the printing process (Figure 1).

The 3D printing equipment used in this study was a Bio-Architect^®^ SR (Hangzhou Regenovo Biotechnology Co., Ltd., Hangzhou, China) 3D printer. After multiple rounds of experimental optimization, the nozzle size and printing parameters were determined as follows: a nozzle diameter of 0.41 mm, a layer height of 0.32 mm, a printing speed of 10 mm/s, and an air pressure of 0.3 MPa. Subsequently, the designed models were printed using the ceramic slurry to obtain ceramic green bodies, which were then subjected to debinding and sintering. The debinding process was carried out as follows: first, the samples were heated to 300 °C at a rate of 1 °C/min and held for 1 h; then, they were heated to 600 °C at 2 °C/min and held for 2 h; finally, the ceramic samples were furnace-cooled to room temperature to complete the debinding process. The high-temperature sintering procedure was as follows: the samples were first heated to 300 °C at 1 °C/min and held for 1 h, then heated to 600 °C at 2 °C/min and held for 1 h, followed by heating to 1550 °C at 2 °C/min and holding for 2 h. Finally, the ceramic samples were furnace-cooled to room temperature to complete the sintering process, yielding sintered ceramic. Table 1 lists the basic mechanical properties of the sintered ceramics tested in this study, which serve as a reference for the subsequent analysis.

### 2.2. Design and Preparation of Biomimetic Samples

The dorsal surface of the desert scorpion is extensively exposed to wind-blown sand and has the largest contact area with sand-laden airflow, making it the primary region subjected to sand erosion. Therefore, in this study, the dorsal surface of the desert scorpion was selected as the biomimetic prototype to guide the design of a biomimetic erosion-resistant structure.

**Figure 2:**
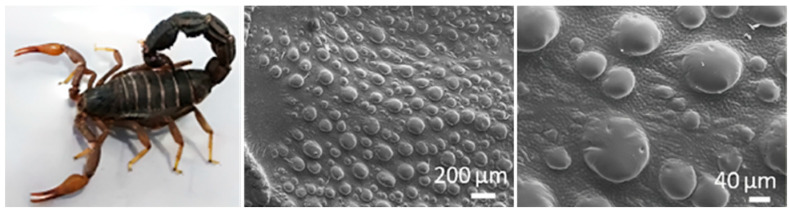
The overall morphology and micro-scale convex bumps of the desert scorpion [31].

As shown in Figure 2, the dorsal plate of the desert scorpion is covered with micron-scale convex bumps arranged non-uniformly [31]. This feature alters the trajectory of sand particles and enhances the erosion resistance of the organism, thereby serving as an active defense strategy against sand erosion. Considering practical processing constraints and the required fabrication precision, the radius of the convex bumps was set to 2.5 mm in this study. Their arrangement was simplified into two configurations: aligned and staggered. The overall dimensions of the model were 30 mm × 30 mm × 5 mm, with an inter-bump spacing of 2 mm. Erosion wear tests were conducted on the two types of biomimetic samples with convex-hump morphologies. The results revealed that, across all tested impact angles, the samples with an aligned arrangement consistently exhibited a lower erosion rate than those with a staggered arrangement. Therefore, the aligned convex-bump samples were selected as the primary focus of this study.

As a macroscale structural coupling element with relatively large feature dimensions, the dorsal plate of the desert scorpion exhibits a certain degree of curvature [20]. During sand particle erosion, this curved structure also serves to modify the actual impact angle of the particles and deflect their trajectories. Morphology–structure coupled biomimetic ceramic composites with curved surfaces were fabricated by DIW 3D printing. Erosion tests were conducted to investigate the effect of the curved structure on the erosion resistance of the convex-bump-based biomimetic sample.

The dorsal plate of the desert scorpion is not composed of a single hard material; rather, it is a composite structure with a hardness gradient, consisting of a rigid outer chitinous layer and a soft inner elastic protein layer [32]. The rigid layer resists damage caused by the impact and cutting of sand particles, while the flexible layer further enhances erosion resistance by absorbing and dissipating the kinetic energy of the impacting particles. In this study, silicone rubber (Shenzhen Sinwe Electronic Material Co., Ltd., Shenzhen, China) with a low elastic modulus was used as the flexible layer. When combined with the convex-curved surface sample, it forms a biomimetic ceramic composite that couples rigid–flexible material properties with morphological and structural features. The dimensional parameters of the rigid layer in the convex rigid–flexible coupled biomimetic model were consistent with those of the convex-curved surface sample. The flexible layer had an edge height of 3 mm, and its length and width matched the lower edge of the rigid layer, ensuring tight bonding between the two layers.

The fabrication of the convex rigid–flexible coupled biomimetic sample involved a two-step process. First, the convex-curved surface biomimetic sample was fabricated by DIW 3D printing. After debinding and sintering, a sintered ceramic rigid layer was obtained. Next, the flexible layer was fabricated directly using a mold. During preparation of the flexible layer, an appropriate amount of removable adhesive was first evenly applied to the inner side of the upper cover and bonded to the surface of the rigid layer, thereby fixing the rigid layer in position. Subsequently, an appropriate amount of silicone rubber was slowly and uniformly injected into the material chamber, and the upper cover was placed at the designated position, allowing the sample to be formed in a closed space. After the silicone rubber had fully cured, the mold was disassembled. Finally, excess silicone rubber along the outer edge of the sample was trimmed off to obtain a rigid–flexible coupled biomimetic sample.

**Figure 3:**
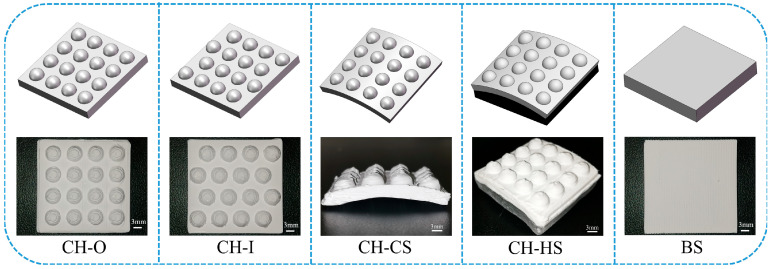
Fabrication of biomimetic ceramic composite samples by 3D printing.

Figure 3 shows the fabricated biomimetic ceramic composite samples. For clarity in the following discussion, the samples are designated as follows in this study: the aligned convex-bump biomimetic sample is denoted as CH-O; the staggered convex-bump biomimetic sample as CH-I; the convex-bump/curved-surface coupled biomimetic sample as CH-CS; the convex-bump hybrid rigid–flexible coupled biomimetic sample as CH-HS; and the smooth control sample as BS.

### 2.3. Erosion Wear Test

**Figure 4:**
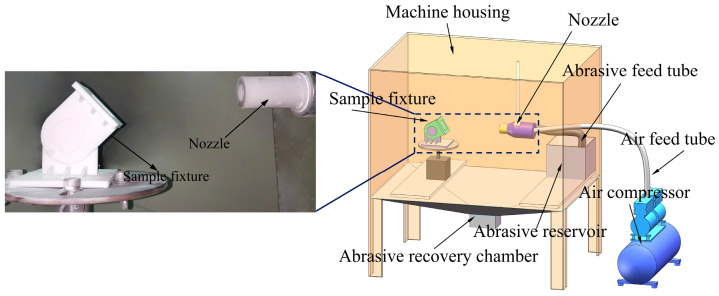
Schematic diagram of the erosion testing machine.

In this study, gas–solid two-phase erosion wear tests were conducted on the biomimetic ceramic samples using a particle-blast erosion wear tester in accordance with ASTM G76-18 [33]. Figure 4 shows a schematic illustration of the test apparatus. The abrasive blasting system consisted of a nozzle for directing abrasive particles and high-pressure gas toward the sample; an abrasive feed tube for delivering the erosive particles; an air feed tube for supplying high-pressure gas; an abrasive reservoir for storing the erosive particles; and an air compressor for providing the required erosion pressure. Additionally, the equipment includes an abrasive recovery chamber for collecting used particles after erosion, and a sample clamping platform for securing test specimens and adjusting the impact angle.

Solid particle erosion wear behavior is influenced by multiple factors, primarily including: the erosion conditions [34,35], such as erosion temperature, particle impact velocity, and erosion duration; the intrinsic properties of the samples [36], including surface morphology and surface roughness; and the characteristics of the abrasive particles [17,37], such as material type, size, shape, and hardness. This study primarily focuses on investigating the effects of biomimetic factors and impact angle on the erosion wear behavior of ceramic composites. Therefore, all other experimental parameters were kept constant.

Al_2_O_3_ ceramics are typical brittle materials, for which the maximum erosion rate occurs at an impact angle close to 90°. Therefore, 90° was selected as one of the impact angles. Under room-temperature erosion conditions, ZTA ceramics exhibit erosion wear behavior similar to that of ductile materials, for which the maximum erosion rate typically occurs at an impact angle of around 20°. Therefore, 15° was selected as one of the impact angles. With 15° and 90° defined as the lower and upper limits of the impact angle range, and using 15° intervals, additional angles of 30°, 45°, 60°, and 75° were selected to investigate the erosion wear behavior of different biomimetic ceramic composites at various impact angles. Additionally, silica sand (primarily composed of SiO_2_) was selected as the erosive particle in this study. Its Vickers hardness is higher than that of the self-prepared ZTA ceramics; therefore, the erosion tests were conducted under conditions in which the erosive particles were harder than the target material. The specific experimental conditions are summarized in Table 2.

Each sample was subjected to erosion for 40 s. First, the sample was placed on the sample stage, and the impact angle was adjusted. Next, the erosive particles were loaded into the abrasive reservoir, the pressure was set on the air compressor control panel, and the erosion duration was set on the equipment control unit. Finally, the spray gun was activated to initiate the erosion test. Every 10 s, the sample was removed from the fixture, cleaned in an ultrasonic cleaner, and weighed using an analytical balance to record its mass. Subsequently, the sample was reinstalled into the fixture. The erosion resistance of the sample was quantified based on the ratio of the material mass loss to the abrasive mass consumption per unit time, and was calculated using the following formula:

(1) $$\delta =\frac{m_{1}-m_{2}}{m}$$

In this equation, *δ* denotes the erosion wear rate (mg/g); m_1_ is the initial mass of the sample (mg); m_2_ is the mass of the sample after erosion (mg); and m is the mass of abrasive particles consumed per unit time (g).

To minimize experimental error, the erosion wear rate for each type of sample was determined as the average of three repeated tests conducted under identical conditions. To investigate the erosion-resistant mechanisms of the biomimetic samples, the surface morphologies of the samples before and after the erosion tests were characterized using a scanning electron microscope (SEM; SEM3100, CIQTEK Co., Ltd., Hefei, China).

## 3. Results and Discussion

### 3.1. Erosion Wear Results and Analysis

**Figure 5:**
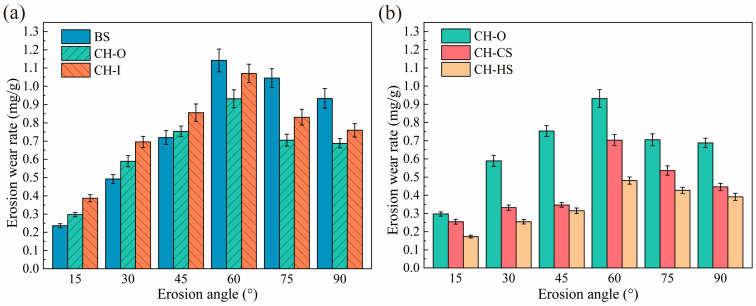
Erosion results at different impact angles: (**a**) biomimetic convex-bump samples; (**b**) coupled biomimetic samples.

Figure 5 shows the variation in erosion rate with impact angle for the biomimetic samples and the control sample throughout the entire erosion process. It can be seen that the erosion rate is strongly dependent on the impact angle. As the impact angle increased from 15° to 90°, the erosion rate of all samples first increased and then decreased, with the minimum erosion rate occurring near 15° and the maximum near 60°. As mentioned above, the addition of 8Y-TZP may have imparted a certain degree of plasticity to the Al_2_O_3_ ceramic, which originally exhibited the behavior of a brittle material. Consequently, the maximum erosion rate of all the ceramic materials tested did not occur at 90°.

The CH-O sample exhibited a lower erosion rate than the CH-I sample at all impact angles (Figure 5a). Erosion tests conducted at impact angles of 15°, 30°, and 45° were classified as low-angle erosion tests, whereas those conducted at 60°, 75°, and 90° were classified as high-angle erosion tests. In the low-angle erosion tests, the erosion rates of the CH-O sample at 15°, 30°, and 45° were approximately 1.26, 1.20, and 1.05 times those of the BS sample at the corresponding impact angles, respectively. In the high-angle erosion tests, the CH-O sample exhibited a lower erosion rate than the BS sample, with erosion resistance improved by 18.39%, 32.54%, and 26.34% at 60°, 75°, and 90°, respectively, relative to the BS sample. The CH-O sample exhibited lower erosion resistance than the BS sample in the low-angle erosion tests, but significantly higher erosion resistance under high-angle erosion conditions.

As shown in Figure 5b, the variation in erosion rate with impact angle for the coupled biomimetic samples is presented. In the low-angle erosion tests, the CH-CS sample exhibited improvements in erosion resistance of 14.31%, 43.73%, and 53.92% at 15°, 30°, and 45°, respectively, compared with the CH-O sample. Meanwhile, relative to the CH-CS sample, the CH-HS sample showed further improvements in erosion resistance of 32.16%, 23.34%, and 9.22% at 15°, 30°, and 45°, respectively. In the high-angle erosion tests, the CH-CS sample exhibited improvements in erosion resistance of 24.57%, 23.97%, and 35.17% at 60°, 75°, and 90°, respectively, compared with the CH-O sample. Meanwhile, relative to the CH-CS sample, the CH-HS sample showed further improvements in erosion resistance of 31.58%, 20.34%, and 12.33% at 60°, 75°, and 90°, respectively.

A comprehensive analysis indicates that the improvement in erosion resistance achieved through biomimetic design strongly depends on the impact angle. Compared with the smooth control sample, CH-O improved erosion resistance by 18.39%, 32.54%, and 26.34% at 60°, 75°, and 90°, respectively, indicating that the protective effect of the convex-bump morphology became more pronounced under high-impact-angle conditions. In contrast, CH-CS further improved the erosion resistance of CH-O by 14.31–53.92% at low impact angles and by 24.57–35.17% at high impact angles, suggesting that the curved-surface design contributed to improved erosion resistance over a broader range of impact angles. CH-HS exhibited a further improvement of 9.22–32.16% over CH-CS, confirming the effectiveness of rigid–flexible coupling in further mitigating erosion damage.

**Figure 6:**
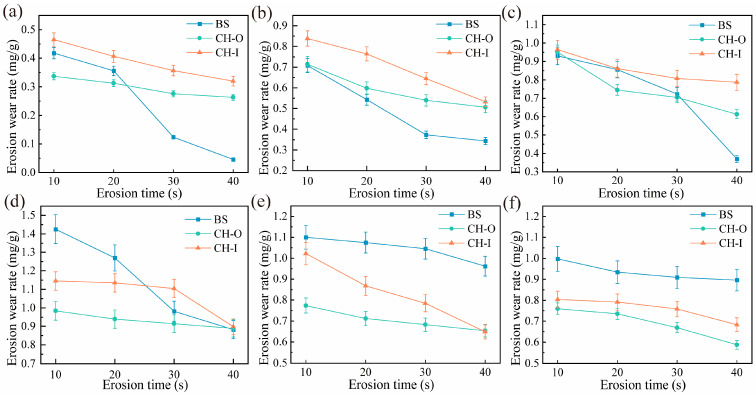
Erosion rate of bionic convex-bump sample as a function of erosion time: (**a**) 15°; (**b**) 30°; (**c**) 45°; (**d**) 60°; (**e**) 75°; (**f**) 90°.

Figure 6 shows the variation in erosion rate with erosion time for the biomimetic convex-bump samples. As can be seen from the figure, the erosion rates of the biomimetic convex-bump samples generally exhibited a continuous decreasing trend with increasing erosion time, indicating that the surface morphology of the samples gradually stabilized and became better adapted to abrasive particle impact. In the low-angle erosion tests, the erosion rates of the biomimetic convex-bump samples remained higher than those of the BS sample throughout the entire erosion process, suggesting that the surface morphology of the biomimetic samples struggled to reach a relatively stable state under low-angle erosion conditions. In the high-angle erosion tests, however, the biomimetic convex-bump samples exhibited excellent erosion resistance and a greater ability to reach a relatively stable state than the BS sample.

**Figure 7:**
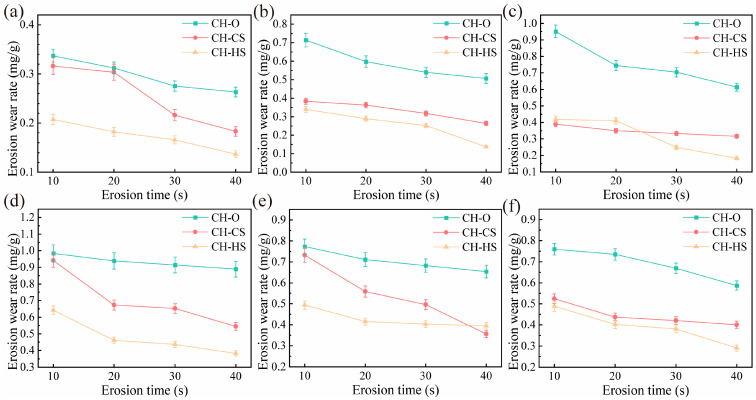
Erosion rate evolution in coupled biomimetic samples over erosion time: (**a**) 15°; (**b**) 30°; (**c**) 45°; (**d**) 60°; (**e**) 75°; (**f**) 90°.

Figure 7 shows the variation in erosion rate with erosion time for the coupled biomimetic samples. The time-dependent erosion behavior of the CH-CS sample indicates that its erosion rate gradually decreased with increasing erosion time, suggesting that the surface morphology of the CH-CS sample evolved toward a state better adapted to the particle erosion environment, thereby enabling the material to reach a relatively stable state. Similarly, the erosion rate of the CH-HS sample also exhibited a decreasing trend with increasing erosion time, indicating that its surface morphology gradually stabilized. At multiple time intervals, the erosion rates of both the CH-CS and CH-HS coupled biomimetic samples were lower than that of the CH-O sample, demonstrating that the curved-surface structure and flexible material enabled the samples to achieve a relatively stable surface morphology more rapidly.

### 3.2. Erosion Morphology Analysis

**Figure 8:**
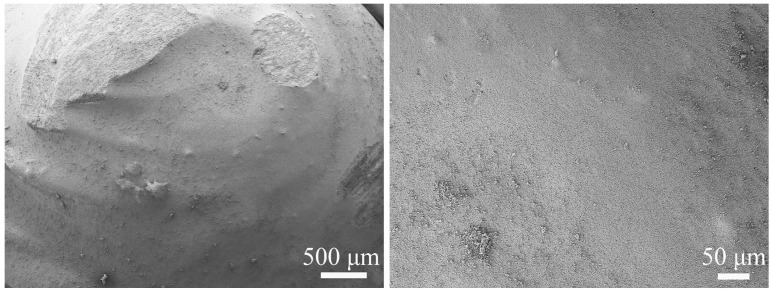
Original surface morphology of the convex bump.

**Figure 9:**
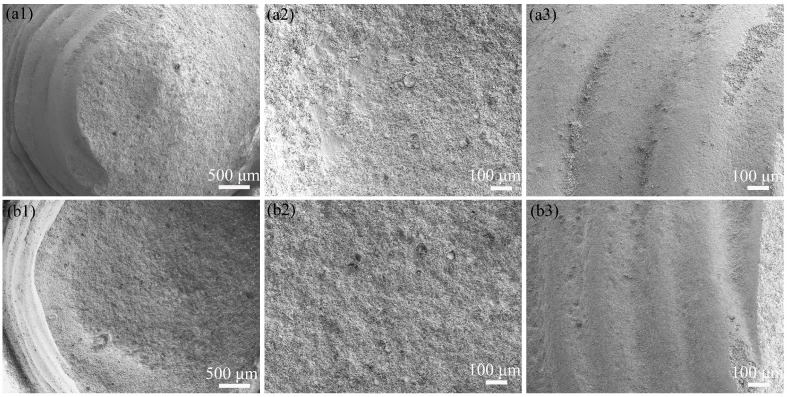
(**a1**) 30° erosion morphology; (**a2**) 30° erosion windward zone; (**a3**) 30° erosion leeward zone; (**b1**) 60° erosion morphology; (**b2**) 60° erosion windward zone; (**b3**) 60° leeward zone.

Figure 8 shows the original surface morphology of the biomimetic convex-bump sample. It can be seen that the convex-bump surface is dense and relatively smooth, with clearly visible printing layer lines. To further elucidate the erosion-resistant mechanisms of the biomimetic sample surfaces, the microscopic morphologies of the eroded biomimetic convex-bump samples were examined at impact angles of 30° and 60°. As shown in Figure 9, under erosive conditions, the surface of the biomimetic convex-bump sample exhibited distinct erosion zones, including a windward zone and a leeward zone. In the windward zone, numerous erosion pits formed as a result of particle impact, leading to increased surface roughness. The printing layer lines were no longer visible, indicating that the surface morphology had undergone substantial changes. In contrast, the leeward side experienced much less severe erosion damage. The printing layer lines remained clearly visible, and the material surface retained relative smoothness and density.

After impacting the material surface, the eroded particles may rebound and re-impact the surface due to gravity or collisions with other particles, resulting in secondary erosion. Although secondary erosion also causes material damage, the kinetic energy of the particles is substantially reduced after their initial impact on an inclined surface. Consequently, the energy available for subsequent impact on another inclined surface is greatly reduced. This may be one of the reasons why the leeward zone of the biomimetic convex-bump sample experiences relatively minor erosion damage.

**Figure 10:**
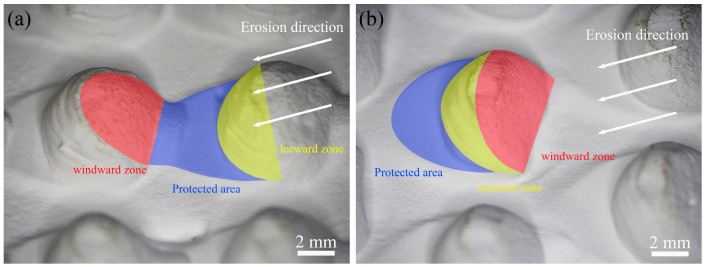
Macroscopic surface of the biomimetic convex-bump samples: (**a**) CH-O sample; (**b**) CH-I sample.

Observation of the biomimetic convex-bump samples revealed a protected area on the leeward zone. As shown in Figure 10, a small, raised platform with relatively low erosion wear was identified between the windward and leeward zones of two adjacent convex bumps along the erosion direction. Because this platform resembles the shadow cast by a convex bump under backlighting, this phenomenon is referred to as the “shadow effect.” The formation of such a “shadow” indicates that the convex bumps obstruct the trajectories of some of the erosive particles, thereby preventing widespread damage to the substrate surface.

### 3.3. Actual Impact Angle Analysis

The angle between the impact velocity direction of the solid particles and the sample surface is referred to as the impact angle, which plays a crucial role in the erosion process [38,39]. For flat samples, the impact angle of solid particles equals the angle between the airflow direction and the sample surface. However, the presence of biomimetic convex protrusions significantly alters the actual impact conditions. When solid particles strike these convex features, the impact angle substantially influences their interaction with the sample surface.

**Figure 11:**
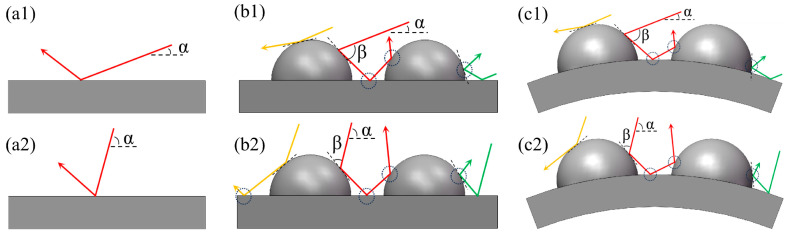
Actual impact angle of biomimetic samples: (**a1**) low-angle actual impact angle of BS sample; (**a2**) high-angle actual impact angle of BS sample; (**b1**) low-angle actual impact angle of CH-O sample; **(b2**) high-angle actual impact angle of CH-O sample; (**c1**) low-angle actual impact angle of CH-CS sample. (**c2**) high-angle actual impact angle of CH-CS sample.

The convex-bump morphology exhibits a strong ability to modify the actual impact angle of the particles and deflect their incident trajectories. As shown in Figure 11, in the low-angle erosion tests, the actual impact angle *β*, defined as the angle between the particle trajectory and the outer surface of the convex bump, increased with increasing particle incident angle *α*. At the same time, the deflection effect of the convex-bump morphology on the particles gradually weakened, leading to stronger direct particle impact on the CH-O sample. As a result, the CH-O sample exhibited lower erosion resistance than the BS sample. In the high-angle erosion tests, however, the trend in the actual impact angle differed. The actual impact angle *β* between the particle trajectory and the convex surface decreased as the particle incident angle *α* increased. In this case, the convex-bump morphology more effectively deflected the incoming particles, thereby continuously reducing the direct impact on the CH-O sample. Consequently, under the same test conditions, the CH-O sample exhibited superior erosion resistance to the BS sample.

The convex-bump morphology of the CH-CS sample continued to modify the actual impact angle of the particles during erosion. In the low-angle erosion tests, the actual impact angle *β* was greater than the particle incident angle *α*, resulting in more severe erosion damage to the material. In the high-angle erosion tests, however, the actual impact angle β was smaller than the particle incident angle α, leading to comparatively less severe erosion wear. Furthermore, after the introduction of the curved surface, the originally flat substrate adopted an upward-curved profile, causing the entire sample to function as a larger-scale protrusion. When the abrasive particle flow impacted the arched substrate surface, the curved structure deflected the particle trajectories, causing them to rebound away from the sample surface and thereby reducing direct impact. It is through the synergistic effect of the large-scale curved structure and the small-scale convex-bump morphology that the CH-CS sample achieved superior erosion resistance compared with the convex-bump sample.

### 3.4. Stress-Wave Propagation Mechanism Analysis

A stress wave is defined as a continuous disturbance of stress and strain within a material that typically propagates in the form of waves. The propagation of stress waves across the interface between different media is governed by fundamental material properties such as elastic modulus, density, and geometry, resulting in transmitted and reflected waves. The magnitudes of these disturbances are determined by the wave impedance of the materials [40]. Table 3 summarizes the acoustic impedances of the rigid and flexible materials used in this study, as calculated using the following formula:

(2) $$\rho C=\sqrt{E\rho }$$

In this equation, *ρC* denotes the acoustic impedance, *E* the elastic modulus of the material, and *ρ* the material density.

Because this study only analyzes the stress-wave propagation mechanism under idealized physical conditions, the analysis is limited to one-dimensional normal elastic waves, specifically the case of normal incidence in which the external stress is perpendicular to the layer interfaces. When an elastic wave propagating through one material in a layered structure reaches the interface with another material, disturbances are generated in both media. Assuming perfect interfacial contact between the two materials, the incident disturbance Δ*σ_in_*, reflected disturbance Δ*σ_re_*, and transmitted disturbance Δ*σ_tr_* satisfy the following relationship:

(3) $$\left\{\begin{array}{l} \Delta \nu_{in}+\Delta \nu_{re}=\Delta \nu_{tr}\\ \Delta \sigma_{in}+\Delta \sigma_{re}=\Delta \sigma_{tr} \end{array}\right.$$

According to the law of momentum conservation, the above equation can be rewritten as follows:

(4) $$\frac{\Delta \sigma_{in}}{{\left(\rho C\right)}_{1}}+\frac{\Delta \sigma_{re}}{{\left(\rho C\right)}_{1}}=\frac{\Delta \sigma_{tr}}{{\left(\rho C\right)}_{2}}$$

By combining the above two equations, we obtain:

(5) $$\left\{\begin{array}{l} \Delta \sigma_{re}=F\cdot \Delta \sigma_{in}\\ \Delta \nu_{re}=-F\cdot \Delta \nu_{in}\\ \Delta \sigma_{tr}=T\cdot \Delta \sigma_{in}\\ \Delta \nu_{tr}=nT\cdot \Delta \sigma_{in} \end{array}\right.$$

In this equation, the wave impedance ratio is defined as *n* = (*ρC*)_1_/(*ρC*)_2_; the reflection coefficient is *F* = (1 − *n*)/(1 + *n*); and the transmission coefficient is *T* = 2/(1 + *n*). Here, *T* is always greater than zero, whereas the sign of *F* depends on the relative magnitudes of the wave impedances of the two media in the layered structure. Specifically, the following two cases apply:

When (*ρC*)_1_ < (*ρC*)_2_, *F* > 0. In this case, the incident and reflected disturbances share the same sign, indicating that the stress wave propagates from the flexible layer to the rigid layer within the layered structure, and results in reflection loading. As (*ρC*)_2_ approaches infinity, *n* approaches zero, *F* approaches one, and *T* approaches two, representing stress wave reflection at a fixed end.

When (*ρC*)_1_ > (*ρC*)_2_, *F* < 0. In this case, the incident and reflected disturbances have opposite signs, indicating that the stress wave propagates from the rigid layer to the flexible layer within the layered structure, and results in reflection unloading. As (*ρC*)_2_ approaches zero, *n* approaches infinity, *F* approaches one, and *T* approaches two, representing stress wave reflection at a free end.

In this study, the acoustic impedance of the rigid layer in the CH-HS sample was significantly higher than that of the flexible layer. When particles impacted the hard surface layer of the sample, the stress wave propagated from the rigid medium into the flexible inner layer, and reflection unloading occurred at the interface. The flexible layer, with its lower acoustic impedance, effectively cushioned and dissipated the impact energy of the erosive particles [41]. Meanwhile, the design parameters of the rigid layer in the CH-HS sample were derived from those of the CH-CS sample; thus, it also inherited the particle-trajectory deflection and actual impact-angle modification effects conferred by its specialized surface morphology during the erosion process. In summary, the CH-HS sample integrates a synergistic biomimetic design incorporating convex-bump morphology, curved-surface architecture, and rigid–flexible coupled materials. This configuration enables the coupling of multiple erosion-resistant mechanisms, leading to a pronounced enhancement in erosion resistance.

## 4. Conclusions

Guided by biomimetic principles, natural forms, structures, and materials often evolve toward configurations that provide better performance with lower energy consumption. In this study, the desert scorpion—renowned for its exceptional erosion resistance—was selected as the biomimetic prototype. Key biological features, including convex bumps, curved surface structures, and heterogeneous layered architecture, were extracted to establish a biomimetic model incorporating both morphological and structural characteristics. The effects of these biomimetic factors on the erosion wear behavior of ceramic composites were investigated through gas–solid two-phase erosion wear tests conducted at various impact angles. The main conclusions are as follows:

(1) The convex-bump morphology modifies the actual impact angle on the biomimetic sample, preventing particles from striking the surface at their original incident angle. This provides effective protection to the material in the leeward region. Compared with the control sample, the biomimetic convex-bump sample exhibited superior erosion resistance in the high-angle erosion tests.
(2) Both the curved-surface structure and the flexible material enhanced the erosion resistance of the biomimetic sample surface. The erosion test results at identical impact angles showed a successive improvement in erosion resistance from the convex-bump biomimetic sample to the convex-bump curved-surface biomimetic sample and finally to the convex-bump rigid–flexible coupled biomimetic sample.
(3) The coupling of biomimetic convex-bump morphology, curved-surface structure, and flexible material significantly enhances the erosion resistance of the ceramic composite. The flexible material cushions and dissipates the kinetic energy of the impacting particles, while the rigid layer with biomimetic morphological characteristics deflects particle trajectories and modifies their actual impact angles. The curved structure causes the originally flat substrate to arch upward, forming a larger-scale ridge-like feature that enables the sample to reach a relatively stable surface state more rapidly. Through the synergistic action of these multiple erosion-resistant mechanisms, the convex-bump rigid–flexible coupled biomimetic ceramic composite achieves a more pronounced improvement in erosion resistance.

## Figures and Tables

**Table 1:** Basic physical properties of sintered ceramics.

Basic Performance	Specific Parameters
Density (g/cm^3^)	4.70 ± 0.4
Linear shrinkage (%)	Length: 6.25 ± 0.4; Width: 6.52 ± 0.5; Height: 6.72 ± 0.5
Bending strength (MPa)	126.657 ± 10.3
Fracture Toughness (MPa·m^1/2^)	2.702 ± 0.2
Elastic modulus (MPa)	26,204 ± 2123

**Table 2:** Specific parameters of erosion test.

No.	Test Conditions	Specific Parameters
1	Particle material	Quartz sand
2	Particle shape	Irregular
3	Particle size (μm)	100–200
4	Erosion pressure (MPa)	0.5
5	Airflow velocity (m/s)	30
6	Particle supply rate (g/s)	25
7	Erosion angle (°)	15, 30, 45, 60, 75, 90
8	Erosion time (s)	40
9	test temperature (°C)	Room temperature
10	Nozzle inside diameter (mm)	8
11	Distance between nozzle and sample (mm)	15

**Table 3:** Wave impedance of the experimental materials.

Material	Elastic Modulus (MPa)	Density (g/cm^3^)	Wave Impedance (MPa·mm^−1^·s^−1^)
ZTA ceramic	26,204	4.7	11.10
Silicone rubber	8.2	1.36	0.11

## Data Availability

The data presented in this study are included in the article. Further inquiries can be directed to the corresponding author.

## References

[1] Zhai W., Bai L., Zhou R., Fan X., Kang G., Liu Y., Zhou K. (2021). Recent Progress on Wear-Resistant Materials: Designs, Properties, and Applications. Adv. Sci..

[2] Hussain Z., Wang X., Riaz I., Lin Z., Nie W., Lin Y., Tao C., Huang Y. (2026). Experimental and multi-scale simulation evaluation of nanoclay/nanosilica epoxy coatings for enhanced corrosion protection of structural steel. Corros. Sci..

[3] Wang X., Cao Q., Tang F., Pan H., Chen X., Lin Z. (2023). Mechanical Properties and Corrosion Behavior of Dual-Filler-Epoxy-Coated Steel Rebar under a Corrosive Environment. Coatings.

[4] Zhang W. (2022). A novel ceramic with low friction and wear toward tribological applications: Boron carbide-silicon carbide. Adv. Colloid Interface Sci..

[5] Ayode Otitoju T., Ugochukwu Okoye P., Chen G., Li Y., Onyeka Okoye M., Li S. (2020). Advanced ceramic components: Materials, fabrication, and applications. J. Ind. Eng. Chem..

[6] Zhao B., Liu H., Huang C., Wang J., Cheng M. (2017). Theoretical hardness analysis and experimental verification for composite ceramic tool materials. Ceram. Int..

[7] Ramachandran K., Bear J.C., Jayaseelan D.D. (2025). Oxide-Based Ceramic Matrix Composites for High-Temperature Environments: A Review. Adv. Eng. Mater..

[8] Ma S., Huang Q., Fei J., Yan J., Zhang T., Li H. (2025). Low-cost and rapid preparation of high-performance carbon fiber reinforced ceramic matrix composites by molding-in situ densification. Ceram. Int..

[9] Zoli L., Servadei F., Bassi G., Rossi A., Montesi M., Vinci A., Sciti D., Panseri S. (2024). From outer space to inside the body: Ultra-high temperature ceramic matrix composites for biomedical applications. J. Eur. Ceram. Soc..

[10] Rocha-Rangel E., la Fuente A.P.-d., Rodríguez-García J.A., Estrada-Guel I., Martínez-Sánchez R. (2017). Effect of silver nanoparticles on the microstructure and mechanical properties of alumina ceramics. Can. Metall. Q..

[11] Liu F.-J., Fang M.-H., Huang Z.-H., Liu Y.-G., Huang S.-F., Min X., Hu M.-L., Ji H.-P. (2012). Preparation and mechanical properties of NiCr–Al_2_O_3_–ZrO_2_(8Y) ceramic composites. Mater. Sci. Eng. A.

[12] Amirthan G., Udayakumar A., Bhanu Prasad V.V., Balasubramanian M. (2010). Solid particle erosion studies on biomorphic Si/SiC ceramic composites. Wear.

[13] Shi W., Tan Y., Hao J., Li  J. (2018). Effect of precoated carbon layer on microstructure and anti-erosion properties of SiC coating for 2D-C/C composites. Int. J. Appl. Ceram. Technol..

[14] Chen Y., Wu Y., Hong S., Long W., Ji X. (2020). The effect of impingement angle on erosion wear characteristics of HVOF sprayed WC-Ni and WC-Cr3C2-Ni cermet composite coatings. Mater. Res. Express.

[15] Han Z., Mu Z., Yin W., Li W., Niu S., Zhang J., Ren L. (2016). Biomimetic multifunctional surfaces inspired from animals. Adv. Colloid Interface Sci..

[16] Sun F., Xu H. (2020). A review of biomimetic research for erosion wear resistance. Bio-Des. Manuf..

[17] Zhang S., Zhang J., Zhu B., Niu S., Han Z., Ren L. (2020). Progress in Bio-inspired Anti-solid Particle Erosion Materials: Learning from Nature but Going beyond Nature. Chin. J. Mech. Eng..

[18] Kumar R., Rezapourian M., Rahmani R., Maurya H.S., Kamboj N., Hussainova I. (2024). Bioinspired and Multifunctional Tribological Materials for Sliding, Erosive, Machining, and Energy-Absorbing Conditions: A Review. Biomimetics.

[19] Yu H., Shao L., Zhang S., Zhang J., Han Z. (2022). An innovative strategy of anti-erosion: Combining bionic morphology and bionic arrangement. Powder Technol..

[20] Han Z., Zhu B., Yang M., Niu S., Song H., Zhang J. (2017). The effect of the micro-structures on the scorpion surface for improving the anti-erosion performance. Surf. Coat. Technol..

[21] Zhang S., Zhang J., Yu H., Niu S., Lian Z., Xu J., Han Z., Ren L. (2023). Large-area preparation strategy and anti-erosion mechanism for morphology-material coupled biomimetic anti-erosion functional surface. Wear.

[22] Zhang F., Zhou S., You H., Zhang G., Yang J., Shi Y. (2025). 3D printing of ceramic matrix composites: Strengthening and toughening strategies. Compos. Part B Eng..

[23] Bose S., Akdogan E.K., Balla V.K., Ciliveri S., Colombo P., Franchin G., Ku N., Kushram P., Niu F., Pelz J. (2024). 3D printing of ceramics: Advantages, challenges, applications, and perspectives. J. Am. Ceram. Soc..

[24] Wu Y., Lan J., Wu M., Zhou W., Zhou S., Yang H., Zhang M., Li Y. (2024). Rheology and Printability of a Porcelain Clay Paste for DIW 3D Printing of Ceramics with Complex Geometric Structures. ACS Omega.

[25] Kafkaslıoğlu Yıldız B., Yıldız A.S., Kul M., Tür Y.K., Işık E., Duran C., Yılmaz H. (2024). Mechanical properties of 3D-printed Al_2_O_3_ honeycomb sandwich structures prepared using the SLA method with different core geometries. Ceram. Int..

[26] Song C., Chen Y., Liu Z.,  Li Y., Yang Y., Yu J. (2024). Rapid and high-accuracy forming of ceramic parts by DLP technology based on optimization of shear stress. J. Eur. Ceram. Soc..

[27] Zhao Q., Chen R., Wang S., Hao W., Dong W., Li X., Wang L. (2024). Utilization of fused deposition modeling in the fabrication of lattice structural Al_2_O_3_ ceramics. Ceram. Int..

[28] Tan W.K., Kuwana T., Yokoi A., Kawamura G., Matsuda A., Muto H. (2021). Electrostatically assembled SiC–Al_2_O_3_ composite particles for direct selective laser sintering. Adv. Powder Technol..

[29] Saadi M.A.S.R., Maguire A., Pottackal N.T., Thakur M.S.H., Ikram M.M., Hart A.J., Ajayan P.M., Rahman M.M. (2022). Direct Ink Writing: A 3D Printing Technology for Diverse Materials. Adv. Mater..

[30] Shahzad A., Lazoglu I. (2021). Direct ink writing (DIW) of structural and functional ceramics: Recent achievements and future challenges. Compos. Part B Eng..

[31] Zhang J., Chen W., Yang M., Chen S., Zhu B., Niu S.,  Han Z., Wang H. (2017). The Ingenious Structure of Scorpion Armor Inspires Sand-Resistant Surfaces. Tribol. Lett..

[32] Han Z., Feng H., Yin W., Niu S., Zhang J., Chen D. (2015). An Efficient Bionic Anti-Erosion Functional Surface Inspired by Desert Scorpion Carapace. Tribol. Trans..

[33] (2018). Standard Test Method for Conducting Erosion Tests by Solid Particle Impingement Using Gas Jets.

[34] Fang M., Liu F., Min X., Huang Z., Liu Y., Wu X., Tang C., Zhang L., Peng F. (2015). Effect of temperature on solid particle impact erosion wear mechanism of 5mol% Yttria Stabilized Zirconia ceramics. Ceram. Int..

[35] Yang S.-Q., Fan J.-C., Liu M.-T., Li D.-N., Li J.-L., Han L.-H., Wang J.-J., Yang S.-Y., Dai S.-W., Zhang L.-B. (2024). Research on the solid particle erosion wear of pipe steel for hydraulic fracturing based on experiments and numerical simulations. Pet. Sci..

[36] Liu R., Pan Y., Chen A., Bin G., Li H. (2023). Study on the influence of surface roughness on the erosion characteristics of compressor blades. Powder Technol..

[37] Singh J., Kumar S., Mohapatra S.K. (2018). Study on role of particle shape in erosion wear of austenitic steel using image processing analysis technique. Proc. Inst. Mech. Eng. Part J J. Eng. Tribol..

[38] Fidan S., Sınmazçelik T., Ürgün S. (2022). Effect of particle flow direction in particle erosion of macro texturized polymer surfaces. Prog. Addit. Manuf..

[39] Jung S., Yang E., Jung W., Kim H.-Y. (2018). Anti-erosive mechanism of a grooved surface against impact of particle-laden flow. Wear.

[40] Wang L., Wu C., Fan L., Wang M. (2022). Effective velocity of reflected wave in rock mass with different wave impedances of normal incidence of stress wave. Int. J. Numer. Anal. Methods Geomech..

[41] Zhang Y., Huang H., Ren L. (2014). Erosion wear experiments and simulation analysis on bionic anti-erosion sample. Sci. China Technol. Sci..

